# RNACompress: Grammar-based compression and informational complexity measurement of RNA secondary structure

**DOI:** 10.1186/1471-2105-9-176

**Published:** 2008-03-31

**Authors:** Qi Liu, Yu Yang, Chun Chen, Jiajun Bu, Yin Zhang, Xiuzi Ye

**Affiliations:** 1 Zhejiang California International Nanosystems Institute, Zhejiang University, Hangzhou, 310029, China; 2 College of Life Science, Zhejiang University, Hangzhou, 310027, China; 3 James D. Watson Institute of Genomic Science, Zhejiang University, Hangzhou, 310008, China; 4 College of Computer Science, Zhejiang University, Hangzhou, 310027, China; 5 SolidWorks Company, MA, USA

## Abstract

**Background:**

With the rapid emergence of RNA databases and newly identified non-coding RNAs, an efficient compression algorithm for RNA sequence and structural information is needed for the storage and analysis of such data. Although several algorithms for compressing DNA sequences have been proposed, none of them are suitable for the compression of RNA sequences with their secondary structures simultaneously. This kind of compression not only facilitates the maintenance of RNA data, but also supplies a novel way to measure the informational complexity of RNA structural data, raising the possibility of studying the relationship between the functional activities of RNA structures and their complexities, as well as various structural properties of RNA based on compression.

**Results:**

*RNACompress *employs an efficient grammar-based model to compress RNA sequences and their secondary structures. The main goals of this algorithm are two fold: (1) present a robust and effective way for RNA structural data compression; (2) design a suitable model to represent RNA secondary structure as well as derive the informational complexity of the structural data based on compression. Our extensive tests have shown that *RNACompress *achieves a universally better compression ratio compared with other sequence-specific or common text-specific compression algorithms, such as *Gencompress, winrar *and *gzip*. Moreover, a test of the activities of distinct GTP-binding RNAs (aptamers) compared with their structural complexity shows that our defined informational complexity can be used to describe how complexity varies with activity. These results lead to an objective means of comparing the functional properties of heteropolymers from the information perspective.

**Conclusion:**

A universal algorithm for the compression of RNA secondary structure as well as the evaluation of its informational complexity is discussed in this paper. We have developed *RNACompress*, as a useful tool for academic users. Extensive tests have shown that *RNACompress *is a universally efficient algorithm for the compression of RNA sequences with their secondary structures. *RNACompress *also serves as a good measurement of the informational complexity of RNA secondary structure, which can be used to study the functional activities of RNA molecules.

## Background

Ribonucleic acid (RNA) is an important class of molecules which performs a wide range of biological and chemical functions. Traditionally, most RNA molecules were regarded as being involved in the process of translation, including transfer RNA (tRNA) and ribosomal RNA (rRNA). Since the late 1990s, it has been widely acknowledged that there exists other type of functional RNA molecules such as non-protein-coding RNAs. These RNAs are found in organisms ranging from bacteria to mammals and affect a wide variety of processes including plasmid replication, phage development, bacterial virulence, chromosome structure, DNA transcription, RNA modification [1-5]. RNA has recently become the center of much attention because of its functions as well as catalytic properties, leading to a substantially increased interest in identifying new RNAs and obtaining their structural information [6-8]. Furthermore, the growth of RNA databases, such as NONCODE [9], Rfam [10], RNaseP [11] and RNAdb [12] has increased two to three fold annually.

To facilitate the maintenance and analysis of such RNA data, an efficient compression algorithm of RNA sequences is needed. Algorithms for compressing DNA sequences include *GenCompress *[13], *DNACompress *[14], *Biocompress *[15] and *Cfact *[16]. However, these algorithms are only suitable for compressing the primary sequences of DNA. As for RNA sequences, we are more interested in designing a novel compression algorithm to compress RNA primary sequence together with its secondary structure information. RNA secondary structure is similar to an alignment of nucleic acid sequences, except that the sequence folds back on itself and "complementary bases" pair (commonly A-U, G-C, G-U) rather than identical or similar bases [17]. The functions of RNA are closely related to its structural characteristics and as such obtaining RNA secondary structure information (both experimentally or computationally) has been an important and interesting problem for several decades [17].

From a strictly mathematical point of view, compression implies understanding and comprehension [18]. Biological sequence compression is a useful tool to recover information from biological sequences. Better compression often implies better understanding. Compressing RNA sequence with secondary structure means that we can capture the essences of RNA sequence information and its structural information simultaneously. From an application point of view, we can derive the informational complexity of RNA structural data based on compression, which can be used to study the structural features and other various properties of RNAs.

In our study, we have developed an efficient grammar-based algorithm to compress RNA sequence and its secondary structure. The software *RNACompress *developed in Windows and Linux platforms is accessible freely at our website. We have also defined the informational complexity of RNA structural data based on compression coupled with the theory of Kolmogorov complexity [18]. This kind of informational complexity will be used to study the relationship between binding activities and structural complexity of RNA aptamers.

To the best of our knowledge, this is the first study to be published about the compression of biological sequences with structural information. Additionally, we apply the results to study functional activities of RNAs. The key idea of our compression algorithm is to use dot-bracket notation [17] to represent the secondary structure of RNA and define specific context free grammars (CFG) to model RNA secondary structure together with its primary sequence during compression (decompression). Furthermore, several computational parser and coding approaches are incorporated to facilitate the whole procedure, including (1) Utilizing the LL(1) parser to derive the left-most derivation of defined grammars for RNA primary sequence and its secondary structure and (2) Using Huffman coding to encode the symbol stream of left-most derivation to achieve the most economical compression result, *etc*. Extensive tests have shown that our algorithm is fast, robust, effective and obtains a universally better compression ratio than the common text-based compression tools or primary-sequence-specific compression tools in the compression of RNA sequence with its structure. These results show that our program is a useful tool for RNA data maintenance and analysis.

## Results

### Algorithm

**Figure 1:**
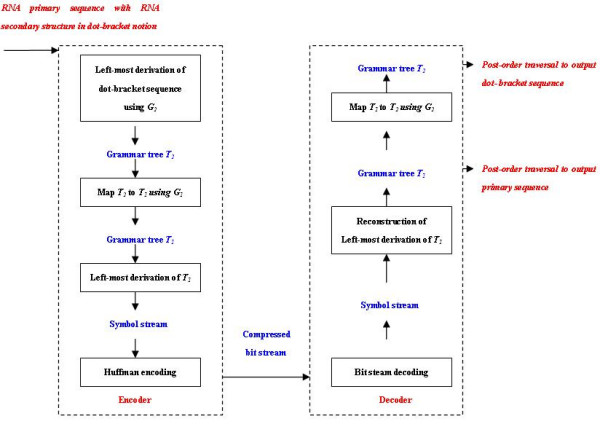
Schema of grammar-based compression and decompression.

Generally speaking, grammar-based compression starts by inferring the context-free grammar to represent the string. The resulting grammar is encoded as a symbol stream, which is then converted into binary. Each step affects the final size of the compressed file. In our algorithm, each step will be designed specifically to facilitate the particular goal of RNA sequence and structure compression. The main schema of our grammar-based compression and decompression is shown in Figure 1. Two specific grammars *G*_1 _and *G*_2 _are defined in our study: *G*_1 _is viewed as the key grammar to represent RNA primary sequence together with its secondary structure, while *G*_2 _is only used to model the dot-bracket sequence of RNA secondary structure and serves as a complementary to *G*_1 _to guide its generation order (As will show later). We start with parsing the dot-bracket sequence in *G*_2 _using the *left-most deriving *[19] to get a grammar tree *T*_2_. At the same time, each deriving step is mapped to construct another grammar tree *T*_1 _based on *G*_1_. Finally this *left-most deriving *symbol stream of *T*_1 _is encoded using Huffman coding theory [20], so that the probability of each unpaired bases and base pairs occurring in the whole secondary structure is considered to get the most economic coding result. As for decompression, the reverse procedure is performed. It should be noted that once the grammar tree *T*_1 _or *T*_2 _is regenerated during decompression, the corresponding primary sequence and secondary structure in dot-bracket notation can be regained using the *post-order traversal *[19] of the leaves of these grammar trees, respectively. More details will be presented in the following.

#### A. Content free grammars of RNA sequence and structure

**Figure 2:**
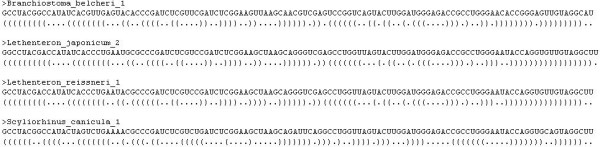
An example of input file for compression.

In our study, RNA primary sequence is represented in FASTA format beginning with a single-line description or comment, followed by lines containing sequence data. The description line is distinguished from the sequence data by a greater-than (">") symbol in the first column. For each sequence, the corresponding secondary structure is represented in dot-bracket notation. Dot-bracket notation is the dominant RNA secondary structure format. It uses dot to represent un-paired bases and brackets to represent base pairs in RNA stems. Many useful tools use this format as input (and output) and hence it has become an unofficial standard [21,22]. As for our compression, the dot-bracket notation was proved to be an efficient way to represent RNA secondary structure and suitable for our grammar parser. A simple example of our input file for compression is shown in Figure 2.

We have defined two concise content free grammars *G*_1 _and *G*_2 _to model RNA primary sequence and its secondary structure information. A CFG is very similar to a finite automaton [23], and has been proved to be an efficient model to study RNA secondary structure. It contains the following elements, which are defined as follows:

(1). Terminals – a symbol that represents a constant value

(2). Non-terminals – a symbol that has the capability of being further defined in terms of terminals and/or non-terminals, usually denoted by a capital letter.

(3). Production rules – rules by which non-terminals can be replaced.

In our study, two grammars are defined as:

*G*_1_:

S: LS | e

L: aSu | uSa | cSg | gSc | uSg | gSu | a| u| c| g

*G*_2_:

S: LS | e

*L: (S) | *•

For both grammars, *S *and *L *are non-terminals, *e *is empty string, and the symbols *a*, *u*, *c*, *g*, (,) and • are terminals representing the 4 different bases, left bracket, right bracket and dot, respectively.

**Figure 3:**
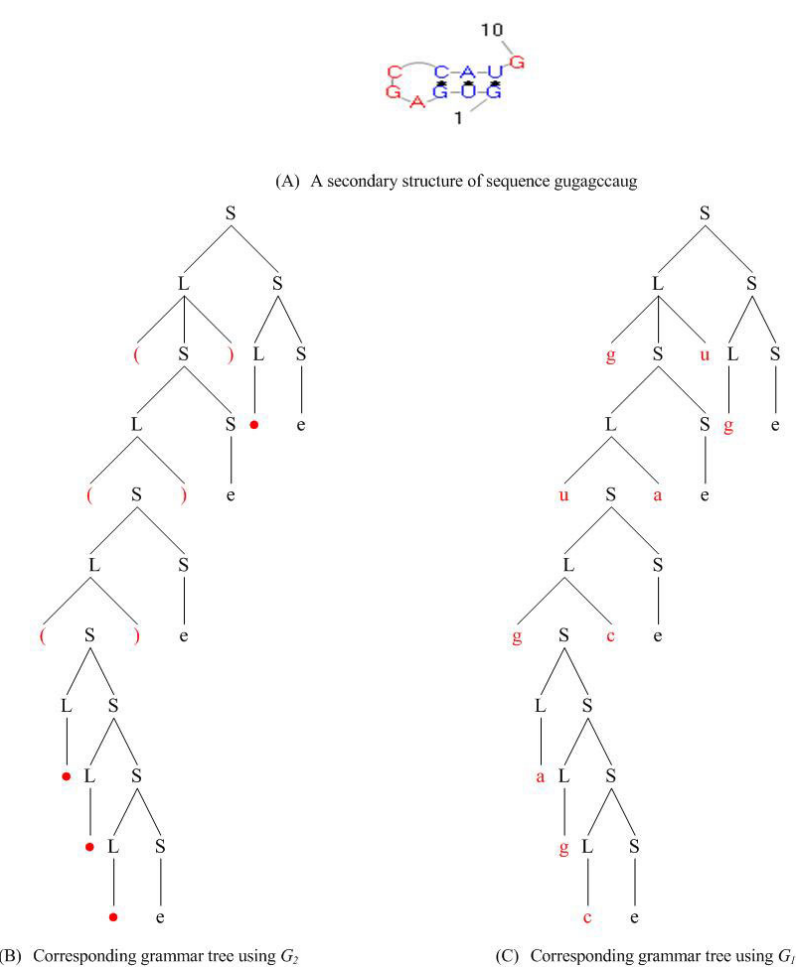
Two kinds of grammar tree of RNA sequence gugagccaug with the corresponding dot-bracket notation (((...))).

Here *G*_1 _is a combination grammar to analyze RNA primary sequence and secondary structure simultaneously. It can model Watson-Crick base pairs A-U, G-C and Wobble base pair G-U in RNA secondary structure. *G*_2 _is aimed at modeling the dot-bracket sequence of RNA secondary structure. With these two grammars, two kinds of grammar trees can be generated for the RNA primary sequence and secondary structure, respectively, as shown in Figure 3. It should be noted that *G*_1_is ambiguous, meaning that the same primary sequence can be generated with more than one grammar tree, while *G*_2 _is unambiguous which means that one dot-bracket string corresponding to only one grammar tree [19]. Thus we have utilized *G*_2 _to guide *G*_1_during the grammar parsing to identify the grammar tree of *G*_1_.

#### B. Compression algorithm

Based on the two grammars we have defined, we are able to perform the compression as shown in Figure 1. In the following we also take the RNA sequence in Figure 3 as an example to demonstrate the whole compression procedure. First we discuss several computational approaches used in our work.

#### LL(1) parser

We start from parsing the dot-bracket sequence of RNA secondary structure using *G*_2_, and the *LL(1) *parser is used to derive the *left-most derivation *of the input sequence. A *LL *parser is a top-down parser for a subset of the context-free grammars [24]. It parses the input from left to right, and constructs a *left-most derivation *of the sentence. Practically, there are two common ways to describe how a given string can be derived from the start symbol of a given grammar. The simplest way is to list the consecutive strings of symbols, beginning with the start symbol and ending with the string, and the rules that have been applied. If we introduce a strategy such as "always replace the left-most non-terminal first" then for context-free grammars the list of applied grammar rules is by itself sufficient. This is defined as the *left-most derivation *of a string [19].

As for *LL(1) parser*, it uses one token of *look-ahead *when parsing a sentence. The parser consists of:

(1) an input buffer, a string from the grammar

(2) a stack on which to store the terminals and non-terminals from the grammar yet to be parsed.

(3) a parsing table which tells it what (if any) grammar rule to apply given the symbols on top of its stack and the next input token.

In our study, the parser applies the rule in the parser table (Table 1) which we have defined for grammar inference of RNA secondary structure, by matching the top-most symbol on the stack (row) with the current symbol in the input stream (column). When the parser starts, the stack already contains two symbols: [S, #], where '#' is a special terminal to indicate the bottom of the stack and the end of the input stream, and 'S' is the start symbol of the grammar. The parser will attempt to rewrite the contents of this stack to what it sees on the input stream. Three types of steps for our *left-most derivation *are followed depending on whether the top of the stack is a non-terminal, a terminal or the special symbol #:

**Table 1:** LL(1) parser table for dot-bracket sequence of RNA secondary structure.

	**(**	**)**	**•**	**#**
**S**	*S:LS*	*S:e*	*S:LS*	*S:e*
**L**	*L:(S)*	*\*	*L: *•	*\*

Each cell in the table gives the production rules used when matching the top-most symbol on the stack (row) with the current symbol in the input stream (column).

(1) If the top of the stack is a non-terminal symbol, the non-terminal symbol and the symbol on the input stream is looked up in the parsing table to determine which rule of the grammar to use. The number of the rule is written to the output stream. If the parsing table indicates that there is no such rule then it reports an error and stops.

(2) If the top of the stack is a terminal symbol, then it is compared to the symbol on the input stream. If they are equal they are both removed. If they are not equal, the parser reports an error and stops.

(3) If the top is # and on the input stream there is also a # then the parser reports that it has successfully parsed the input, otherwise it reports an error. In both cases the parser will stop.

These steps are repeated until the parser stops, and then it will have either completely parsed the input or written a *left-most derivation *to the output stream or it will have reported an error.

#### Map left-most derivation of G_2 _to G_1_

As mentioned above, *G*_2 _is used to guide the *left-most derivation *of *G*_1 _since it is ambiguous. The mapping of the *left-most derivation *of *G*_2 _to *G*_1 _is straightforward: '()' will be mapped to the corresponding base pairs of the RNA secondary structure and '•' will be mapped to the corresponding un-paired bases. After this mapping, a *left-most derivation *of *G*_1 _is obtained and the Huffman coding is performed on the symbol stream of this left-most derivation to encode them into a bit stream, as discussed follow.

#### Huffman coding

Huffman coding is an entropy encoding algorithm used for lossless data compression. The term refers to the use of a variable-length code table for encoding a source symbol where the variable-length code table has been derived in a particular way based on the estimated probability of occurrence for each possible value of the source symbol [20]. Huffman coding is able to design the most efficient compression method of this type: no other mapping of individual source symbols to unique strings of bits produces a smaller average output size when the actual symbol frequencies agree with those used to create the code.

We use variable-length code table to encode the symbol stream of *left-most derivation *of *G*_1 _based on the probability associated for each production rules. *G*_1 _can be viewed as a stochastic context free grammar (SCFG) [25]. We have derived the rule probabilities based on a complete statistic analysis of the frequency distribution of base pair and un-paired bases in different RNA secondary structure using the RNA structural database *RNABase *[26]. Nearly 1200 RNA sequences which cover diverse RNAs including tRNA, rRNA, non-coding RNA *etc*. are examined and the final statistical probabilities are listed in Table 2.

**Table 2:** Huffman coding of production rules of grammar G_1_

**Production rules**	**Probability**	**Huffman code**
*S: LS*	/	0
*S:e*	/	1
*L: a*	0.183076	00
*L: u*	0.158666	100
*L: c*	0.087876	1111
*L: g*	0.101709	010
*L: aSu*	0.071603	0110
*L: uSa*	0.094386	1110
*L: cSg*	0.144020	110
*L: gSc*	0.113914	101
*L: uSg*	0.026851	01110
*L: gSu*	0.017901	01111

It should be noted that for different types of RNA or RNA in different species, the frequency distribution of their base pairs or un-paired bases are different, thus the production probabilities of the rules are different. However, from a statistical perspective, we aim at designing a universal compression algorithm for all types of RNA, thus we make use of these general probabilities here. For more specific RNA types, more specific probabilities can be used.

**Figure 4:**
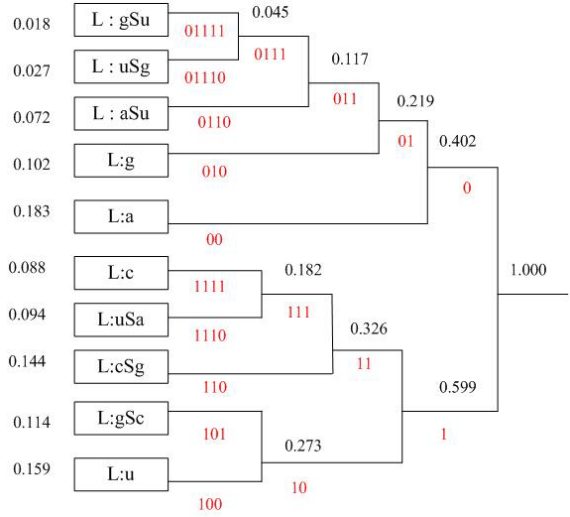
**Huffman coding of production rules of *G*_1_**. This binary tree is generated from left to right taking the two least probable symbols and putting them together to form another equivalent symbol having a probability that equals the sum of the two symbols. The process is iterated until we have only one symbol. Finally, the tree is read backwards, from right to left, to assign different bits to different branches.

The Huffman tree based on our statistical probabilities is shown in Figure 4. Finally variable-length bit codes are generated to encode the different production rules.

#### Example

We took the RNA sequence in Figure 3 as an example to demonstrate the whole compression procedure, as shown in Table 3. The input is a RNA primary sequence with its secondary structure in dot-bracket notation. Each step of the *LL(1) *parser and the corresponding operation is also listed.

**Table 3:** An example of compression

**Step**	**Stack**	**Input buffer**	**Production Rules of *G*_2_**	**Map to *G*_1_**	**Huffman code**
1	# S	(((...))).#	*S:LS*	*S:LS*	0
2	# S L	(((...))).#	*L:(S)*	*S:gSu*	01111
3	# S) S (	(((...))).#			
4	# S) S	((...))).#	*S:LS*	*S:LS*	0
5	# S) S L	((...))).#	*L:(S)*	*S:uSa*	1110
6	# S) S) S (	((...))).#			
7	# S) S) S	(...))).#	*S:LS*	*S:LS*	0
8	# S) S) S L	(...))).#	*L:(S)*	*S:gSc*	101
9	# S) S) S) S (	(...))).#			
10	# S) S) S) S	...))).#	*S:LS*	*S:LS*	0
11	# S) S) S) S L	...))).#	*L: *•	*L:a*	00
12	# S) S) S) S .	...))).#			
13	# S) S) S) S	..))).#	*S:LS*	*S:LS*	0
14	# S) S) S) S L	..))).#	*L: *•	*L:g*	010
15	# S) S) S) S .	..))).#			
16	# S) S) S) S	.))).#	*S:LS*	*S:LS*	0
17	# S) S) S) S L	.))).#	*L: *•	*L:c*	1111
18	# S) S) S) S .	.))).#			
19	# S) S) S) S	))).#	*S:e*	*S:e*	1
20	# S) S) S)	))).#			
21	# S) S) S	)).#	*S:e*	*S:e*	1
22	# S) S)	)).#			
23	# S) S	).#	*S:e*	*S:e*	1
24	# S)	).#			
25	# S	.#	*S:LS*	*S:LS*	0
26	# S L	.#	*L: *•	*L:g*	010
27	# S .	.#			
28	# S	#	*S:e*	*S:e*	1
29	#	#			

The final bit stream of this left-most derivation is 0 01111 0 1110 0 101 0 00 0 010 0 1111 1 1 1 0 010 1, for a total of 35 bits.

#### Definition of compression ratio

In our work, the compression ratio of the compression algorithm can be computed in two ways:

*R*_1 _= *uncompressed_file_bytesize/compressed_file_bytesize*, or *R*_2 _= (*n *× (*H*_1 _+ *H*_2_))/*o*, where *n *is the number of the bases in input RNA sequence. *H*_1 _and *H*_2 _are the information entropy of the RNA primary sequence and secondary structure, respectively. *o *is the number of bits in compressed file.

While *R*_1 _is a straightforward way to compute the compression ratio based on the byte size of the input file and the compressed file, *R*_2 _is more specific based on entropy theory. In the definition of *R*_2_, the uncompressed input file is divided into two parts: RNA primary sequence and RNA secondary structure in dot-bracket notation. From an information entropy perspective [27], since there exists four bases (A, U, G, C) for primary sequence and 3 characters " (", ") " and "." for secondary structure, the average information entropy for the primary sequence (*H*_1_) and secondary structure (*H*_2_) can be computed as:

$$\begin{array}{ccc} H_{1}=-\sum_{1}^{4}P_{i}\log_{2}P_{i} & \text{and} & H_{2}=-\sum_{1}^{3}P_{i}^{'}\log_{2}P_{i}^{'} \end{array}$$

Where *P*_*i *_and *P'*_*i *_are the occurrence probabilities of each bases and characters in dot-bracket notation. If we consider a RNA sequence with infinite length, then *P*_*i *_= 1/4 and *P*'_*i *_= 1/3, assuming an independent probability distribution of 4 base pairs and 3 characters, thus *H*_1 _= 2 and *H*_2 _≈ 1.585. This means that 2 bits is enough for encode the RNA primary sequence and 1.585 bit can be used to encoding RNA secondary structure in dot-bracket notation. Note that in our implementation, the occurrence probabilities of 4 bases and 3 characters will be computed according to the particular RNA.

#### C. Informational complexity

The definition of informational complexity of RNA structural data underlies the concept of Kolmogorov complexity. The Kolmogorov complexity *K*(•) of an object o is defined by the length of the shortest program P for a Universal Turing Machine U that is needed to output o [18]. Intuitively, *K*(*x*) represents the minimal amount of information required to generate × by an algorithm.

It is well known that there is a relationship between Kolmogorov complexity of sequences and Shannon information theory [28]: the expected Kolmogorov complexity of a sequence *x *is asymptotically close to the entropy of the information source emitting *x*. However, Kolmogorov complexity is non-computable in the Turing sense [18] and in practical applications it is approximated by the length of the compressed sequence calculated by a compression algorithm [18].

In summary, the informational complexity of a given RNA sequence with its secondary structure is approximated by the compressed bit string using *RNACompress*. This definition is straightforward, yet with rigorously theoretical support. Later experiment will prove that our informational complexity can reveal the relationship between structural complexity and functional activity of RNA aptamers, which could be useful in predicting the functional utility of novel heteropolymers.

### Experimental testing

Our experiments are performed in two parts: first the compression ability of *RNACompress *is tested, and secondly the results are applied to reveal the relationship between binding activities and structural complexity of RNA aptamers.

#### A. Compression ability

We have tested the compression ability of *RNACompress *on 7 benchmark files that are access freely at our website. These 7 data files are generated from different databases or curated from literatures, covering diverse types of RNA molecules with their secondary structures (computationally predicted or experimental validate), including rRNA, tRNA and small non-coding RNAs. Note that the compression of one RNA sequence makes no sense from the statistical perspective, since RNA sequence is generally much shorter than the DNA sequences or the whole genomes. In our test, the input file contains a set of RNAs. Also the sequence identities in each input file are different. The intention to use these extensive data files is two folds: to test the behavior of our algorithm in compression of files with different sequence identities and to demonstrate that our algorithm is universally efficient for different type of RNAs. Detail descriptions of these 7 test files are listed in Table 4.

**Table 4:** Descriptions of benchmark data files

**File**	**Type**	**Source**	**Size**	**Description**	**Sequence identities**
*rRNA.txt*	rRNA	5S ribosomal RNA database [34]	10.8 KB	45 metazoan rRNA sequences	High
*tRNA.txt*	tRNA	GtRDB-Genomics tRNA Database	2.06 KB	14 tRNA from various eukaryotes	Medium
*miRNA.txt*	microRNA	miRBase [35]	328 KB	1855 mammalian miRNAs obtained from the latest release of miRBase.	High
*evofold.txt*	Mixed ncRNA	[36]	3.72 MB	47509 functional RNAs identified by Evofold, utilizing a comparative genomics method based on phylogenetic stochastic context-free grammars.	Low
*asRNA.txt*	Mixed ncRNA	[37]	148 KB	97 putative antisense ncRNAs identified from cDNA and EST databases for human and mouse.	Low
*snoRNA.txt*	snoRNA	snoRNA-LBME-db [38]	82.5 KB	411 human snoRNAs and scaRNAs selectedd from snoRNA-LBME-db (release 3, August 2006)	Low
*151Rfam.txt*	Mixed ncRNA	Rfam database [10]	40.7 KB	151 non-coding RNA structures downloaded from Rfam, as collected by Do *et al*. for CONTRAfold training [39]	Low

We have compared the running time and compression ratios (*R*_1 _and *R*_2_) of *RNACompress *with three other algorithms: *Gencompress*, *winrar *and *gzip*. *Gencompress *(*DNACompress *is the newest version of *Gencompress*) has reported to be the top algorithm among all the other sequence-specific compression algorithms, such as *Biocompress *and *Cfact*. The other two algorithms, *winrar *(commercially) and *gzip *(based on Lempel-Ziv coding/LZ77), are two classical text compression algorithm widely used in Windows and Linux, respectively. Detail comparisons between *RNACompress *and these three algorithms are listed in Table 5.

**Table 5:** Comparisons of compression ratios and running times of *RNACompress*, *Gencompress*, *winrar *and *gzip*.

**Test data**	***RNACompress***			***Gencompress***			***Winrar***			***Gzip***		
	
	RT(s)	CR (*R*_1_)	CR (*R*_2_)	RT (s)	CR (*R*_1_)	CR (*R*_2_)	RT(s)	CR (*R*_1_)	CR (*R*_2_)	RT(s)	CR (*R*_1_)	CR (*R*_2_)
*rRNA.txt*	**0.16**	**5.234**	**1.133**	0.55	8.103	1.753	0.13	10.689	2.312	0.11	10.294	2.227
*tRNA.txt*	**0.08**	**5.215**	**1.100**	1.12	4.581	0.964	0.11	4.054	0.853	0.06	4.355	0.917
*miRNA.txt*	**0.24**	**5.248**	**1.126**	316.23	9.171	1.969	0.32	7.847	1.684	0.27	6.176	1.326
*evofold.txt*	**6.56**	**5.252**	**1.046**	/	/	/	4.24	4.302	0.857	2.25	4.241	0.845
*asRNA.txt*	**0.43**	**4.966**	**1.104**	241.36	4.703	1.043	0.41	4.123	0.846	0.32	4.048	0.898
*snoRNA.txt*	**0.52**	**5.073**	**1.089**	71.29	4.778	1.025	0.67	4.324	0.928	0.61	4.113	0.883
*151Rfam.txt*	**0.12**	**4.812**	**1.032**	21.70	4.655	0.997	0.33	3.990	0.850	0.21	3.908	0.837

Tests are performed on Windows and Linux platforms with 512 RAM and 2.0 MHz CPU. (RT: Running Time; CR: Compression Ratio; '/' means that for evofold.txt, Gencompress becomes inapplicable because of the large size of this file.)

It can be seen that *RNACompress *achieves the best compression ratio with comparable speed among the other algorithms, except for two tests file *rRNA.txt *and *miRNA.txt*. For *rRNA.txt*, the sequence identities are nearly 90%. *Gencompress *and other two common compression algorithms are efficient to capture the pattern repeats in this file, thus achieve better results. For *miRNA.txt*, the same reason also holds. Furthermore, microRNAs are generally short RNA molecules of about 21–23 nucleotides in length, thus their ability to be compressed are reduced compared to longer sequences. Although efficient at searching for approximate matches and reverse complements, the running time for *Gencompress *was found to be unpractical long when the input file is large.

Essentially, our compression algorithm is based on grammar inference and Huffman coding, and currently does not consider the repeat patterns of the input file. This is why *RNACompress *failed to achieve the better compression ratio when the sequence identities are high in a set of RNAs. Our algorithm is, however, very robust to different types of RNA and influenced little by the arrangement of the input file. As for the three other algorithms, if we rearrange the same set of RNAs in different order and artificially space out two highly identical sequences, their compression ratios will decrease dramatically. In addition, there also exist other algorithms that are based on different mechanisms besides searching repeat pattern, one of these is PPM [29], which uses a specialized form of compression based on Markov modeling. Unfortunately, these algorithms are generally computation extensive in their exchange for higher compressions.

#### B. Aptamer activity and complexity

To further demonstrate the applications of *RNACompress*, we present a comparison of the structural complexities and activities of RNA aptamers, which was initially conducted by Carothers *et al*.[30]. In their study, a remarkable correspondence between the affinities of eleven GTP-binding RNAs and the intricacy of their secondary structures is found, i.e., aptamers with higher-affinity binding to a target molecule are likely to have more structural informational complexity. However, an efficient calculation of informational complexity was missing in their study. The authors have pointed out the difficult and ambiguity to determine the amount of information of stems in RNA secondary structures and presents three complicated methods to compute it. In our study, we have applied our defined informational complexity to measure the whole structural complexity of RNA aptamers, which makes the calculation more straightforward. Moreover, we have also calculated the Spearman rank correlation coefficient (*r*_*s*_) of the aptamer informational complexity onto the binding activities, as done by Carothers *et al*.. Our results are consistent with their study, which proves that the informational complexity defined here is reasonable when studying the relationship between functional activities and structural complexity of RNA molecules (Table 6). More detail information of eleven GTP-bind RNAs is listed in Additional file 1.

**Table 6:** Spearman Correlation Coefficients (r_s_) of aptamer activity onto the informational complexity.

**Aptamer**	***K*_*d*_(nM)^a^**	**Information content(bits)**			
		
		**Apt(A)^b^**	**Apt(B)^b^**	**Apt(C)^b^**	**Our defined information complexity**
*9-4*	9 ± 1	65.0	56.0	65.0	225
*Class V*	17 ± 4	54.5	44.5	54.5	221
*10-10*	30 ± 6	71.0	65.0	67.0	206
*Class I*	76 ± 3	45.0	41.0	45.0	136
*10-59*	250 ± 20	60.5	53.5	42.5	184
*10-24*	300 ± 50	50.0	44.0	44.0	186
*9-12*	300 ± 50	58.5	54.5	52.5	148
*10-6*	300 ± 100	71.0	65.0	67.0	186
*Class II*	400 ± 200	38.0	36.0	40.0	102
*Class IV*	900 ± 200	36.5	32.5	32.5	142
*Class III*	8000 ± 1000	43.5	38.5	41.5	129
***r*_*s *_of *K*_*d*_**		**0.58**	**0.56**	**0.65**	**0.78**
***P value***		**<0.050**	**<0.050**	**<0.025**	**<0.005**

It can be seen that there is a positive correlation between the aptamer activity and the informational complexity. For more detail information of eleven GTP-bind RNAs, please refer the original paper [30] or the additional file 1.

* K_d_: solution dissociation constant. (Measurement of the affinity activities of GTP-binding RNAs). The smaller the dissociation constant, the more tightly bound the ligand is, or the higher the affinity between ligand and protein. Three different methods for treating the stems were applied to calculate the aptamer information content [30]. These are indicated as Apt (A), Apt (B), and Apt(C).

## Discussion

Generally speaking, if we treat both RNA sequences and the representation of their secondary structures as text, any text-specific compression algorithms can be used to compress them. However, these compressions have no biological meaning and disturb the original RNA structure information, although they may achieve higher compression ratios. From a biological perspective, *RNACompress *is more competitive than any others because it is not only an efficient algorithm to compress RNAs, but also a nice model to represent RNA data. These kinds of compression and representation abilities are based on our grammar inference, which is inherently suitable to capture the structural essence of RNA.

In addition, there still exist several interesting issues in our study, which needs to be discussed or investigated in the future.

(1) currently we are focused on modeling two dominant types of base pairs in RNA secondary structure: Watson-Crick pairs and Wobble pairs. There also exists other minor variations of base-pairing in nucleic acids, such as Hoogsteen base pair (A-T) [31]. One challenge remain problem is how to incorporate the modeling of these minor base pairs and keep the compression ratios simultaneously.

(2) one promising way to improve the compression ability of *RNACompress *is to consider the repeat pattern of RNA motifs in RNA secondary structure. This is different from the repeat pattern identified in primary sequences, as used in *Gencompress etc*. Also it will be helpful to approximate the Kolmogorov complexity and evaluate the informational complexity more accuracy. RNA motifs are basic building blocks used repeatedly, and in various combinations, to form different RNA types and define their unique structural and functional properties. Currently many algorithms for RNA motif identifications have been proposed [6,32,33]. However, these efforts were moderately successfully in define simple RNA structure. A powerful algorithm to capture complex structural domains or various non-canonical pairings in RNA motifs is still needed.

(3) another application of compression RNA secondary structure is that it is a great alignment-free tool for RNA secondary structure comparison. A universal (dis)similarity measure (USM) can be defined to measure the pair-wise distance of RNA secondary structures based on the compression, as we will demonstrate elsewhere (Qi Liu *et al*., RNA secondary structure comparison based on compression: a methodological study, manuscript in preparation).

## Conclusion

In this article we have introduced a universal algorithm for the compression of RNA secondary structure as well as the evaluation of its informational complexity. We have developed *RNACompress*, as a useful tool for academic users. Extensive tests have shown that *RNACompress *is a universally efficient algorithm for the compression of RNA sequences with their secondary structures. *RNACompress *also serves as a good measurement of the informational complexity of RNA secondary structure, which can be used to study the functional activities of RNA molecules. Furthermore, future studies will show that our compression algorithm can facilitate the comparisons of RNA secondary structure and studying of non-coding RNA structures, provides a new way to investigate RNA properties based on compression.

## Availability and Requirements

Project name:

*RNACompress*: Grammar-based compression and informational complexity measurement of RNA secondary structure

**Project home page: **

Operating systems:

Windows 2000/XP and Linux

Programming language:

C/C++

## Authors' contributions

QL carried out the designing of the whole computational algorithm and drafted the manuscript. YY was responsible for the software implementation. YZ and JB were responsible for the data collection and selection. CC and XY conceived the study and participated in the design and coordination of the analyses. All authors read and approved the final manuscript.

## Supplementary Material

Additional file 1 Secondary structures of eleven distinct GTP-binding RNAs (aptamers), sorted by their affinity binding activities. Click here for file
