# Supplementary material for: RNACompress: Grammar-based compression and informational complexity measurement of RNA secondary structure
Source: BMC Bioinformatics. 2008 Mar 31;9:176. doi: 10.1186/1471-2105-9-176 (PMC2335284; doi:10.1186/1471-2105-9-176)
Supplement: Additional file 1 — Secondary structures of eleven distinct GTP-binding RNAs (aptamers), sorted by their affinity binding activities. [file 1471-2105-9-176-S1.doc]

RNACompress: Grammar-based compression and informational complexity measurement of RNA secondary structure

**Supplementary Material**

**Figure. Secondary structures of eleven distinct GTP-binding RNAs (aptamers), sorted by their affinity binding activities.**

**
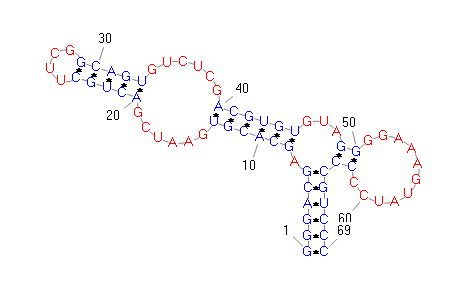
**

**9-4: *K_d_* = 9 nM**

**
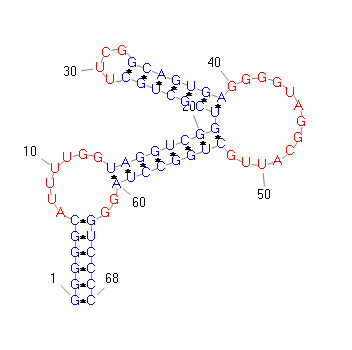
**

**Class V: *K_d_* = 17 nM**

**
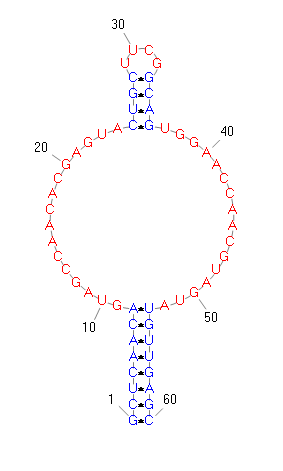
**

**10-10: *K_d_* = 30 nM**

**
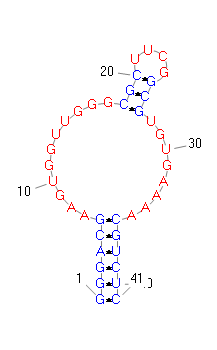
**

**Class I: *K_d_* = 76 nM**

**
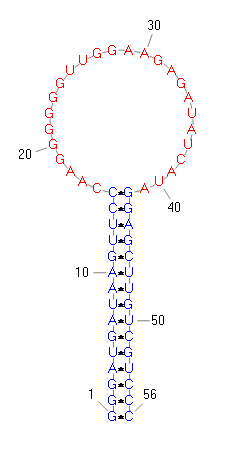
**

**10-59: *K_d_* = 250 nM**

**
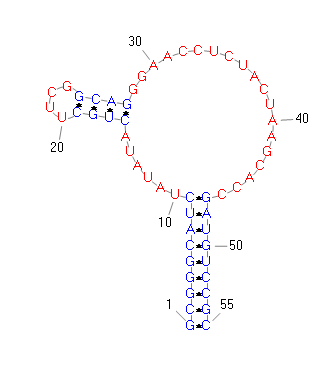
**

**10-24: *K_d_* = 300 nM**

**
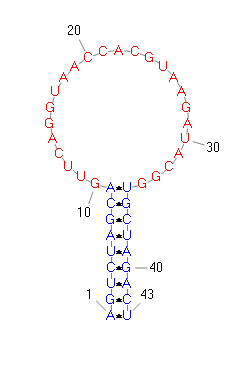
**

**9-12: *K_d_* = 300 nM**

**
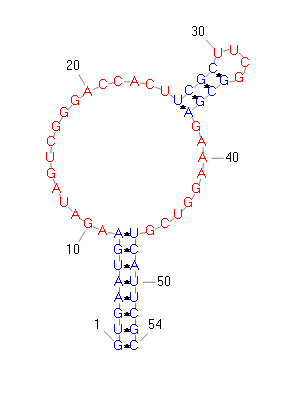
**

**10-6: *K_d_* = 300 nM**

**
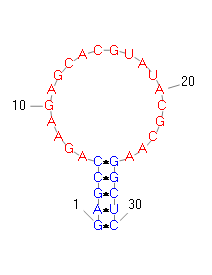
**

**Class II: *K_d_* = 400 nM**

**
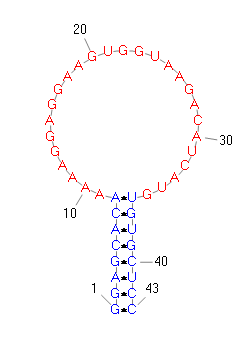
**

**Class IV: *K_d_* = 900 nM**

**
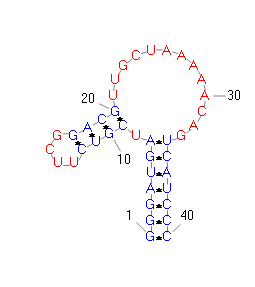
**

**Class III: *K_d_* = 8000 nM**
